# Enhancing Parenteral Nutrition via Supplementation with Antioxidant Lutein in Human Serum Albumin-Based Nanosuspension

**DOI:** 10.3390/pharmaceutics17080971

**Published:** 2025-07-26

**Authors:** Izabela Żółnowska, Aleksandra Gostyńska-Stawna, Katarzyna Dominiak, Barbara Jadach, Maciej Stawny

**Affiliations:** 1 Department of Pharmaceutical Chemistry, Poznan University of Medical Sciences, Rokietnicka 3, 60-806 Poznan, Poland; katarzyna.dominiak@student.ump.edu.pl (K.D.); mstawny@ump.edu.pl (M.S.); 2 Doctoral School, Poznan University of Medical Sciences, Bukowska 70, 60-812 Poznan, Poland; 3 Department of Pharmaceutical Technology, Poznan University of Medical Sciences, Rokietnicka 3, 60-806 Poznan, Poland; bajadach@ump.edu.pl

**Keywords:** carotenoids, xanthophylls, clinical nutrition, nanoparticles, drug delivery systems

## Abstract

**Background/Objectives**: Parenteral nutrition (PN) supports patients unable to receive nutrients via the gastrointestinal tract, but it lacks the health-promoting natural bioactive compounds found in a typical oral diet. This study aimed to develop a human serum albumin-based intravenous delivery system for lutein (an antioxidant carotenoid with vision-supportive and hepatoprotective properties) as a PN additive. **Methods**: An albumin–lutein nanosuspension (AlbLuteN) was synthesized using a modified nanoparticle albumin-bound (nab^TM^) technology and characterized physicochemically. The nanoformulation was added to four commercial PN admixtures to assess the supplementation safety throughout the maximum infusion period. Visual inspection and measurements of fat globules larger than 5 µm (PFAT5) and the mean hydrodynamic diameter (Z-average), zeta potential, pH, osmolality, and lutein content were performed to detect potential interactions and evaluate the physicochemical stability. **Results**: AlbLuteN consisted of uniform particles (Z-average of 133.5 ± 2.8 nm) with a zeta potential of −28.1 ± 1.8 mV, lutein content of 4.76 ± 0.39%, and entrapment efficiency of 84.4 ± 6.3%. Differential scanning calorimetry confirmed the amorphous state of lutein in the nanosuspension. AlbLuteN was successfully incorporated into PN admixtures, without visible phase separation or significant changes in physicochemical parameters. The PFAT5 and Z-average values remained within pharmacopeial limits over 24 h. No substantial shifts in zeta potential, pH, or osmolality were observed. The lutein content remained stable, with losses below 3%. **Conclusions**: AlbLuteN can be safely added to representative PN admixtures without compromising their stability. This approach offers a novel strategy for intravenous lutein delivery and may contribute to improving the nutritional profile of PN.

## 1. Introduction

“You are what you eat” captures the essential role of a balanced diet in maintaining overall health. It is estimated that one in five deaths worldwide could be prevented by improving dietary habits. While healthy eating patterns are crucial in supporting the body’s proper functioning [1], certain groups are unable to consume an oral diet and/or absorb nutrients through the gastrointestinal tract. In such cases, nutrients must be delivered directly into the bloodstream in the form of a nutritional admixture [2].

Glucose, amino acids, lipids, electrolytes, vitamins, and trace elements constitute parenteral nutrition (PN), which is administered as an intravenous (IV) emulsion to patients who cannot maintain adequate nutrition through the oral or enteral route [2]. PN can be utilized both as a short-term intervention in acute cases and as a long-term therapy for chronic illnesses [3]. Intestinal dysfunction that prevents nutrient absorption, caused by conditions like short bowel syndrome, intestinal dysmotility, and mechanical obstruction, is the main indication for prolonged home PN. However, there are patients with a functional gut, including those with cancer, disordered eating, or gastrointestinal neuromuscular disorders, who, for various reasons, also receive IV feeding at home. Such therapy, although beneficial in preventing malnutrition, should be implemented with caution. In clinical practice, long-term parenteral support is not recommended for individuals without intestinal failure who can receive either oral or enteral nutrition, due to the risk of complications associated with prolonged IV feeding [4].

The most common adverse events linked to PN administration include metabolic complications—fat overload syndrome, fatty acid deficiency, and intestinal failure-associated liver disease (IFALD)—as well as infections associated with central venous catheters [5,6]. IFALD is one of the most serious complications, as, in severe cases, it can result in liver failure, with transplantation being the only remaining treatment option. Its pathogenesis is complex. On the one hand, the disease is driven by inflammation-inducing bacterial endotoxins, which enter the bloodstream either following vascular access infections or through a permeable intestinal barrier. On the other hand, liver dysfunction develops due to factors related to the composition of nutritional admixtures, especially the lipid component. An unbalanced omega-3 to omega-6 fatty acid ratio promotes inflammation and liver steatosis, while the high content of phytosterols present in soybean oil used in lipid emulsions exacerbates inflammation and leads to cholestasis. Moreover, PN is deficient in antioxidants that protect against oxidative stress. The plethora of factors aggravating the progression of IFALD requires a multi-pronged approach to its management, which remains a challenge for clinicians. An effective prevention/treatment strategy has yet to be established [7].

Ongoing efforts aim to improve PN and reduce the complications that it causes. One approach involves modifying the composition of lipid emulsions, the most problematic components of PN admixtures. A shift toward next-generation PN formulas, in which emulsions are enriched with different oils to partially replace soybean oil, has made it possible to optimize the lipid fraction [5,7]. While this has somewhat reduced the impact of IV nutrition on the liver, the overall incidence of PN-related adverse events has not decreased following the introduction of multi-oil emulsions. The metabolic complications and infections, as well as less common adverse events affecting the nervous, cardiac, immune, or urinary systems, require further investigation and proper management [5]. Given the persistence of complications, exploring alternative ways to improve the PN composition appears warranted.

PN consists of essential nutrients; however, its composition remains far from that of a balanced diet, which is rich in so-called nonnutritive bioactive substances like polyphenols, terpenoids, and sulfur- or nitrogen-containing compounds that provide significant health benefits [8]. We propose a novel approach to IV nutrition formulation by enriching it with such natural bioactive substances to compensate for their deficiencies in patients who cannot receive nutrition via the gastrointestinal route. This strategy, aiming to render artificial nutrition more similar to an oral diet, has the potential to reduce the incidence of complications associated with PN by supporting overall body function.

We have selected non-provitamin A carotenoids as candidates for PN admixtures enhancement. This is because their structure, characterized by a long chain of conjugated double bonds, enables potent antioxidant activity, and they regulate various signaling pathways at the molecular level [9]. Research links insufficient serum carotenoid concentrations with the development of several chronic diseases and increased all-cause mortality, highlighting the need for supplementation [10]. Among carotenoids, lutein stands out due to its well-documented health benefits. This xanthophyll is abundant in green leafy vegetables, maize, and squash. Alongside zeaxanthin, lutein is the only carotenoid selectively accumulated in the macula of the human retina, where it filters harmful blue light and protects photoreceptor cells from oxidative damage [11]. It is also present in human breast milk, where it can account for up to 50% of the total carotenoids. This accumulation highlights its physiological relevance during early development, as lutein has been suggested to accumulate in brain tissues and play a role in supporting optimal brain health during the initial stages of life [10]. Moreover, lutein accumulates in the liver, where it mitigates oxidative stress, reduces inflammation, and prevents steatosis, underscoring its potential for IFALD prevention and treatment. Studies have shown that lutein exerts its hepatoprotective effects through multiple mechanisms, including the activation of the nuclear factor erythroid 2-related factor 2 signaling pathway, suppression of nuclear factor-κB-mediated inflammatory responses, and modulation of lipid metabolism via the regulation of AMP-activated protein kinase and peroxisome proliferator-activated receptor-α [11]. These mechanisms directly counteract the main drivers of IFALD pathogenesis, such as oxidative stress, chronic inflammation, and hepatic lipid accumulation associated with prolonged PN. Such multi-targeted activity highlights lutein’s potential clinical relevance as a supportive antioxidant in PN regimens [11]. However, lutein’s application is hindered by its poor water solubility, affecting its stability and limiting effective administration [9].

To address this issue and enable the incorporation of lutein into PN admixtures, we aimed to develop a suitable IV carrier system. In this study, we present the preparation and characterization of an albumin–lutein nanosuspension (AlbLuteN)—a human serum albumin-based formulation composed of nanoparticles and lutein–protein complexes. AlbLuteN was prepared using the nanoparticle albumin-bound (nab^TM^) technology, an established platform for the IV delivery of poorly water-soluble active pharmaceutical ingredients (APIs), approved by the Food and Drug Administration (FDA) in formulations such as Abraxane^®^ (nab^TM^ paclitaxel) [12] and Fyarro^®^ (nab^TM^ rapamycin) [13]. This technology eliminates the need for conventional solubilizers and emulsifiers, thereby improving biocompatibility and reducing formulation-related adverse effects. Building on these advantages, the nab^TM^ technology is being increasingly explored as a strategy for the parenteral administration of various poorly soluble APIs [13,14].

We also assessed the stability of four commercially available PN admixtures supplemented with AlbLuteN to ensure its clinical applicability. This study is part of a project aimed at integrating non-provitamin A carotenoids into PN, with further plans focusing on their biological effects and potential benefits for patients suffering from IFALD. To the best of our knowledge, this is the first study on carotenoid supplementation in PN, offering a promising strategy for the reduction of PN-related complications in the future.

## 2. Materials and Methods

### 2.1. Materials

Lutein (pharmaceutical secondary standard) and potassium bromide were obtained from Merck KGaA (Darmstadt, Germany). Albiomin^®^ (20% *w*/*v* human serum albumin solution for IV infusion) was supplied by Biotest Pharma GmbH (Dreieich, Germany). The 0.9% sodium chloride injection and water for injection were procured from B. Braun Melsungen AG (Melsungen, Germany). All solvents used in the study, classified as analytical or high-performance liquid chromatography (HPLC) grade, were purchased from Avantor Performance Materials Poland S.A. (Gliwice, Poland). Tracutil and PN bags (Lipoflex Peri^®^, Omegaflex Peri^®^, Omegaflex Special^®^, and Omegaflex Plus^®^) were manufactured by B. Braun Melsungen AG. Cernevit was obtained from Baxter Polska Sp. z o.o. (Warsaw, Poland).

### 2.2. Preparation of AlbLuteN

The nanosuspension was synthesized using a modified nab^TM^ technology, which involves the preparation of a pre-emulsion followed by the formation of the final emulsion (through high-pressure homogenization) and the evaporation of the organic solvent [13]. In our study, the emulsion was obtained using sonication, omitting the pre-emulsion step.

The synthesis of AlbLuteN commenced with the preparation of 10 mL of a 40 mg/mL human serum albumin solution in a 50 mL narrow beaker by diluting Albiomin^®^ (200 mg/mL) with water for injection. Subsequently, 1 mL of lutein solution in dichloromethane at 25 mg/mL was added to the albumin solution. The beaker was then placed in an ice bath on a magnetic stirrer set to 300 rpm. The mixture underwent sonication using a probe (Sonopuls HD 2070 ultrasonic homogenizer, Bandelin Electronic GmbH & Co. KG, Berlin, Germany) for 3 min at 80% amplitude, employing 15 s treatment intervals with 15 s breaks. The resulting emulsion was transferred to a 100 mL round-bottom flask, and the dichloromethane was evaporated under reduced pressure using a rotary evaporator for 30 min at 40 °C. As the emulsion initially foamed, the pressure was gradually reduced. The nanoparticle suspension was transferred into a vial and freeze-dried according to the conditions described in the literature [15] to obtain a solid form suitable for long-term storage. No cryoprotectant was required. The formulated lyophilizate was kept protected from light at 4 °C for further studies. To reconstitute the freeze-dried cake, water for injection or 0.9% sodium chloride solution was slowly added along the wall of the vial containing the lyophilizate, and it was allowed to hydrate for a few minutes. The vial was then gently rotated to prevent foaming.

The blank nanosuspension was prepared using the same method, except that, at the emulsion preparation stage, pure dichloromethane was used instead of a lutein solution in dichloromethane.

### 2.3. Formulation Characterization

#### 2.3.1. Physicochemical Characterization of AlbLuteN

The physicochemical properties of AlbLuteN were characterized with respect to the particle size distribution, zeta potential, pH, and osmolality. The intensity-weighted mean hydrodynamic diameter (Z-average) and polydispersity index (PDI) were determined by dynamic light scattering using a Zetasizer Nano ZS (Malvern Instruments, Malvern, UK) equipped with a 633 nm laser and DTS1070 cell. Measurements were conducted at 25 °C immediately after sample preparation (post-dichloromethane evaporation) and after the reconstitution of the freeze-dried formulation to assess the colloidal stability over 5 weeks. Samples were stored at 4 °C in the dark under nitrogen. Prior to analysis, they were diluted 100-fold with water for injection.

The zeta potential was measured using the same instrument and sample conditions via laser Doppler electrophoresis, with results calculated according to the Smoluchowski equation.

The pH of AlbLuteN reconstituted in 0.9% sodium chloride was measured with a calibrated Mettler Toledo SevenCompact pH/Ion S220^®^ meter, while osmolality was assessed by cryoscopy using an 800 CL TridentMed osmometer (Trident Med s.c., Warsaw, Poland).

#### 2.3.2. Lutein Crystallinity

The crystallinity of lutein in the nanoformulation, i.e., the determination of whether it was present in a crystalline or amorphous form, was evaluated using differential scanning calorimetry (DSC). The analyses were performed on pure lutein, lyophilized AlbLuteN, a blank nanosuspension, and a physical mixture of the latter with lutein in a quantitative ratio corresponding to the nanoformulation. DSC measurements were conducted using a DSC 214 Polyma differential scanning calorimeter (Netzsch GmbH & Co., Selb, Germany). For each measurement, 5 mg of the sample was sealed in an aluminum pan, with an empty pan serving as the reference. The analysis was performed under a nitrogen purge at a flow rate of 250 mL/min. Samples were heated at a linear rate of 5.0 K/min from 25 °C to 210 °C. The resulting thermograms were analyzed to determine the presence or absence of melting peaks.

#### 2.3.3. Quantitative Analysis

The lutein content in the formulation was determined using HPLC with diode-array detection (DAD). Freeze-dried cakes were homogenized prior to quantitative analysis by grinding in an agate mortar, and the resulting powders were hydrated (10 mg in 1 mL of water). Before column injection, the samples were prepared to enable albumin precipitation and ensure complete lutein dissolution. This involved adding 5 mL of a 1:1 (*v*/*v*) methanol–acetonitrile mixture to 200 µL of the nanosuspension in a centrifuge tube, followed by ultrasonic treatment using a Bransonic CPX3800H-E ultrasonic bath (Branson, Danbury, CT, USA) for 30 min at room temperature. The tubes were then refrigerated for a further 30 min. Subsequently, the samples were centrifuged (Frontier^TM^ 5000 Multi Pro FC5718R centrifuge, Ohaus, NJ, USA) at 15,000 rpm for 15 min at 4 °C. The supernatants were filtered through 0.2 μm syringe filters and analyzed by HPLC-DAD without additional dilution. The concentration of lutein was determined from a matrix-matched calibration curve, performed in triplicate to account for potential matrix effects. For calibration, solutions were prepared in the same manner as for the nanosuspensions, except that blank lyophilizates were used instead of the lyophilized AlbLuteN, and the methanol–acetonitrile mixture contained lutein in various known concentrations.

HPLC-DAD analysis was performed using an Infinity II 1260 system (Agilent Technologies, Santa Clara, CA, USA) equipped with a LiChrospher^®^ 100 RP-18 endcapped column (250 mm × 4 mm, 5 µm particle size) from Merck KGaA (Darmstadt, Germany). Analysis conditions were as follows: injection volume—20 µL; mobile phase composition—methanol–acetonitrile (90:10 *v*/*v*) + triethylamine 9 µM [16]; flow rate—1.5 mL/min; analysis time—6 min; column temperature—30 °C; detection at 475 nm.

Drug loading (*DL%*), i.e., the content of lutein in the formulation, was calculated based on Equation (1):

(1) $$\mathrm{DL}\%\;=\;\frac{\mathrm{mass}\;\mathrm{of}\;\mathrm{lutein}\;\mathrm{in}\;\mathrm{the}\;\mathrm{lyophilizate}}{\mathrm{mass}\;\mathrm{of}\;\mathrm{the}\;\mathrm{lyophilizate}}\;\times \;100.$$

Entrapment efficiency (*EE%*), defined as the effectiveness of incorporating lutein into the nanosuspension, was determined using Equation (2):

(2) $$\mathrm{EE}\%\;=\;\frac{\mathrm{DL}\%\;\mathrm{experimental}}{\mathrm{DL}\%\;\mathrm{theoretical}}\;\times \;100.$$

The API in human serum albumin-stabilized nanosuspensions comes in two forms: encapsulated within the nanoparticles and bound to free albumin in the dispersion [14]. To determine the amount of lutein incorporated into nanoparticles vs. that bound in complexes with free albumin, the two fractions were separated by centrifugation (15,000 rpm, 30 min, 15 °C). The unencapsulated lutein content in the supernatant was determined in parallel with that in non-centrifuged samples according to the procedure described above. Nanoparticle lutein (*NL%*), defined as the percentage of lutein in the formulation encapsulated in nanoparticles, was calculated using Equation (3):

(3) $$\mathrm{NL}\%\;=\;\frac{\mathrm{mass}\;\mathrm{of}\;\mathrm{lutein}\;\mathrm{in}\;\mathrm{the}\;\mathrm{sample}-\mathrm{mass}\;\mathrm{of}\;\mathrm{lutein}\;\mathrm{in}\;\mathrm{the}\;\mathrm{supernatant}}{\mathrm{mass}\;\mathrm{of}\;\mathrm{lutein}\;\mathrm{in}\;\mathrm{the}\;\mathrm{sample}}\;\times \;100.$$

#### 2.3.4. Lutein Encapsulation in Nanoparticles

Fourier transform infrared (FTIR) spectroscopy was used to evaluate the encapsulation of lutein in nanoparticles. The analyzed samples, all in solid form, included pure lutein, AlbLuteN, a blank nanosuspension, and a physical mixture of the blank nanosuspension with lutein, prepared in a ratio reflecting the composition of AlbLuteN. Prior to analysis, 1 mg of each sample was micronized with 300 mg of KBr in an agate mortar and then compressed into a pellet using a hydraulic press. A pure KBr pellet was used for background correction. Spectra were collected using an IRAffinity-IS Fourier transform infrared spectrophotometer (Shimadzu, Kyoto, Japan) in the range of 4000–400 cm^−1^, with a resolution of 4.0 cm^−1^ and an average of 40 scans per spectrum. Following spectral acquisition, baseline correction was performed to minimize background variations, and characteristic bands were identified.

To facilitate the comparison between the test sample (AlbLuteN) and the references (the blank nanosuspension and the physical mixture of the blank with lutein), spectral normalization was performed in absorbance mode based on the area under the curve. The absorbance values of AlbLuteN at specific wavenumbers were then compared separately with those of the blank nanosuspension and the physical mixture. A one-tailed Pearson’s correlation test was performed to assess spectral similarity. The correlation coefficient was determined for the entire spectral range and specifically for the region corresponding to the most prominent lutein band (850–1050 cm^−1^).

Pearson’s correlation coefficient (*r*) was calculated using Equation (4):

(4) $$r=\frac{\sum_{i}^{n}\left(A_{i}-\bar{A}\right)\times (B_{i}-\bar{B})}{\sqrt{\sum_{i}^{n}{\left(A_{i}-\bar{A}\right)}^{2}\times \sum_{i}^{n}{\left(B_{i}-\bar{B}\right)}^{2}}},$$

where *Ai* and *Bi* represent the respective absorbance values at a given wavenumber, *n* is the number of data points, and $\bar{A}$ and $\bar{B}$ denote the average absorbance values of each spectrum.

#### 2.3.5. Nanoparticle Behavior upon Dilution

The size distribution of nanoparticles, upon dilution with water for injection in a simplified non-biological environment, was assessed to evaluate their stability at concentrations relevant to IV administration, following the methodology reported by Yasuda et al. [17]. AlbLuteN samples were initially prepared at a concentration corresponding to 2 mg human serum albumin/mL, and the size was measured without the standard 100-fold dilution prior to analysis. The samples were then sequentially diluted down to a final concentration of 0.05 mg albumin/mL, with a three-minute equilibration period after each dilution before measurement. Size distribution analysis was conducted following the methodology described in Section 2.3.1.

### 2.4. Parenteral Nutrition Stability Study

This study aimed to assess the safety of PN supplementation with AlbLuteN by testing the PN quality after adding our formulation and confirming its stability over the 24 h maximum infusion period. The study design specified the addition of 5 mg of lutein to a 1250 mL PN admixture for an adult patient. Four commercial infusion emulsions were selected—Lipoflex Peri (LPe), Omegaflex Peri (OPe), Omegaflex Special (OSp), and Omegaflex Plus (OPl)—with their characteristics summarized in Table 1. The admixtures were activated by mechanically breaking the seals of their compartments and mixing the contents by repeatedly inverting the bags. They were then supplemented with vitamins (Cernevit, reconstituted according to the manufacturer’s instructions) and trace elements (Tracutil). These admixtures served as controls.

In clinical practice, adding 5 mg of lutein to a complete PN bag would require reconstituting the amount of AlbLuteN lyophilizate containing 5 mg of lutein in 5 mL of 0.9% sodium chloride solution and injecting it into the PN admixture, following the procedure used for vitamin preparations. To replicate these conditions, appropriate volumes of AlbLuteN (1 mg lutein/mL) were added to aliquots of the control admixtures in 10 mL tubes. Three independent samples of AlbLuteN were used for the analyses.

Admixtures were physicochemically characterized immediately after PN preparation and after 24 h of storage at room temperature in the absence of light. Pharmacopeial or literature-based acceptance criteria [18,19] were applied to assess admixture parameters. The admixtures were visually assessed and characterized by determining the percentage of fat residing in globules larger than 5 µm (PFAT5), the Z-average of the droplet size of the emulsion, the zeta potential, pH, and osmolality according to the methodology described before [20].

Lutein content was determined using the same instrumentation and method as described in Section 2.3.3 for AlbLuteN. Due to the complex composition of PN + AlbLuteN samples, appropriate sample preparation was required prior to the quantitative HPLC-DAD analysis of lutein to prevent interference from emulsion lipids and albumin present in the solution. For analysis, 0.5 mL of PN + AlbLuteN was combined with 1 mL of dichloromethane, and the volume was adjusted to 5 mL with methanol. The mixture was then sonicated in the ultrasonic bath at room temperature for 30 min, followed by refrigeration for an additional 30 min and centrifugation at 15,000 rpm for 15 min at 4 °C. The supernatant was filtered through a 0.2 µm syringe filter and injected onto the column without further dilution.

Matrix-matched calibration curves were prepared to determine the lutein content in PN samples supplemented with AlbLuteN. To ensure that the calibration samples closely reflected the composition of the analyzed ones, a blank albumin nanosuspension was added to control emulsions in a volume and concentration corresponding to that of AlbLuteN added to PN in the stability study. For each calibration sample, 0.5 mL of the PN + blank solution was combined with 1 mL of dichloromethane, and the volume was adjusted to 5 mL with methanol, in which lutein was dissolved at various known concentrations. Calibration samples underwent the same preparation steps as the analyzed ones.

Preliminary studies showed that samples containing emulsions with omega-3 fatty acid triglycerides (i.e., all Omegaflex formulations) exhibited no differences in calibration slopes, whereas the sample containing Lipoflex Peri displayed a different slope compared to the Omegaflex formulations, indicating variations in matrix effects. Therefore, two calibration curves were prepared in triplicate—one for Lipoflex Peri and another for Omegaflex Peri, which was used for the evaluation of lutein content in all Omegaflex-containing samples.

### 2.5. Statistical Analysis and Software

All experiments were performed at least three times. Results are expressed as the mean ± standard deviation (SD). A two-tailed Student’s t-test was used for comparisons between two groups, while one-way ANOVA was applied for comparisons among three or more. A *p*-value < 0.05 was considered statistically significant. Statistical analysis was performed using GraphPad Prism v10.4.1. FTIR spectra processing was conducted with Spectragryph v1.2.16.1 [21].

## 3. Results

### 3.1. AlbLuteN Characterization

**Figure 1:**
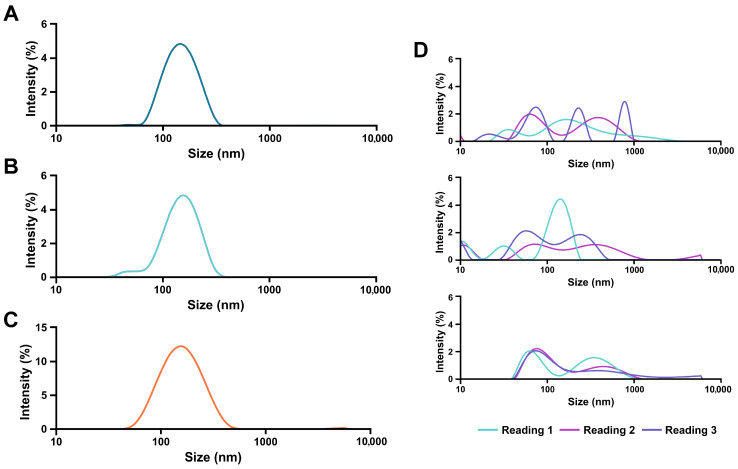
Intensity-weighted size distribution curves obtained by dynamic light scattering. AlbLuteN freshly prepared (**A**), post-reconstitution (**B**), and 5 weeks post-reconstitution (**C**) exhibited uniform particle size distributions. In contrast, blank nanosuspension measurements for three samples (**D**) revealed multiple particle populations with poor reproducibility, both between samples and across individual readings of the same sample.

The nanoparticle size in AlbLuteN was evaluated based on the Z-average and PDI obtained by dynamic light scattering. The Z-average of the freshly prepared nanosuspension was 133.5 ± 2.8 nm, and the initial PDI was 0.194 ± 0.014, indicating particle homogeneity (Figure 1A).

In contrast, the blank nanosuspension exhibited a broad size distribution. The intensity-weighted size distribution curves (Figure 1D) revealed multiple peaks, indicating the presence of particles with varying hydrodynamic diameters. Cumulant analysis, used to determine the Z-average and PDI, assumes a Gaussian particle size distribution centered around a single mean size. Therefore, it was not suitable for obtaining Z-average or PDI values for the blank formulation. Notably, repeated size measurements of the same sample showed variations in peak positions and intensities. This demonstrated that the lack of uniformity in the blank nanosuspensions hindered accurate size analysis. Moreover, differences were observed among the blank sample replicates, highlighting their lack of reproducibility.

**Figure 2:**
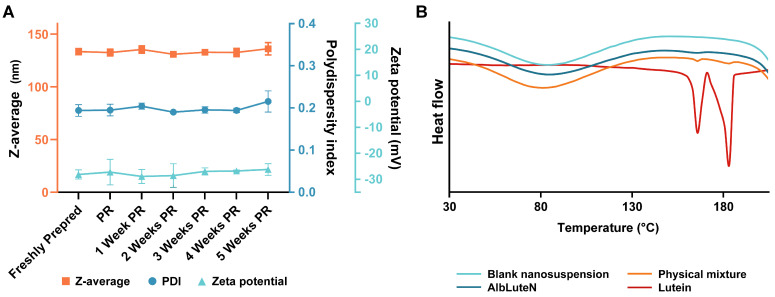
(**A**) Z-average, PDI, and zeta potential values measured in the freshly prepared AlbLuteN, immediately after freeze-dried powder reconstitution, and at weekly intervals post-reconstitution (PR) during the five-week colloidal stability study. No significant differences were observed in these parameters (*p* > 0.05). (**B**) DSC thermograms of lutein, AlbLuteN, the blank nanosuspension, and its physical mixture with lutein. Endothermic peaks are oriented downward. The thermograms represent the first heating cycle.

Since particle homogeneity and stability are crucial for IV formulations, we further evaluated the size distribution of AlbLuteN immediately after post-lyophilization reconstitution and throughout storage. Despite the absence of cryoprotectants, no significant differences were observed in the Z-average (132.6 ± 3.2 nm, *p* = 0.5734) or PDI (0.195 ± 0.014, *p* = 0.9122) immediately after lyophilizate reconstitution compared to the freshly prepared formulation. Similarly, no significant changes in particle diameter or PDI were observed in redispersed AlbLuteN throughout the five-week refrigerated storage period (Figure 2A). The size distribution monitored across the stability study remained narrow (Figure 1B,C).

The zeta potential of AlbLuteN after preparation reached −28.1 ± 1.8 mV. The zeta potential after lyophilizate rehydration did not differ significantly from that of the freshly prepared sample (−27.2 ± 4.9 mV, *p* = 0.8356) and remained stable over the following five weeks of storage at 4 °C, as shown in Figure 2A. The zeta potential of the blank nanosuspension was −8.47 ± 3.89 mV.

The pH and osmolality of AlbLuteN reconstituted in 0.9% saline solution were 6.97 ± 0.01 and 328 ± 2 mOsm/kg, respectively.

DSC was employed to assess the crystalline or amorphous state of lutein in the nanoformulation. Thermograms of pure lutein, lyophilized AlbLuteN, the blank nanosuspension, and the physical mixture of the blank with lutein were recorded and are presented in Figure 2B. The DSC curve of pure lutein exhibited two distinct endothermic peaks at 165 °C and 183 °C. The thermogram of the physical mixture also displayed these characteristic peaks, confirming the presence of crystalline lutein in the sample. In contrast, the lyophilized AlbLuteN did not exhibit these melting peaks, and its thermal profile resembled that of the blank nanosuspension. The absence of lutein’s characteristic melting transitions in the AlbLuteN thermogram suggested that lutein was no longer in its crystalline form within the nanoformulation.

Quantitative HPLC-DAD analysis of lutein in the formulation was performed to determine the *DL%*, *EE%*, and *NL%*. The *DL%*, which represents the proportion of lutein incorporated into the nanosuspension relative to the total nanosuspension mass, was found to be 4.76 ± 0.39%. The *EE%*, reflecting the encapsulation efficiency of lutein based on the initial amount added, reached 84.4 ± 6.3%, indicating an efficient encapsulation process. To assess the distribution of lutein between the encapsulated and free albumin-bound fractions, the nanosuspension was subjected to centrifugation, followed by the quantification of lutein in the supernatant. The *NL%* was determined to be 51.4 ± 2.9%, indicating that roughly half of the total lutein was encapsulated within nanoparticles, while the remaining fraction was bound to free albumin in the dispersion.

**Figure 3:**
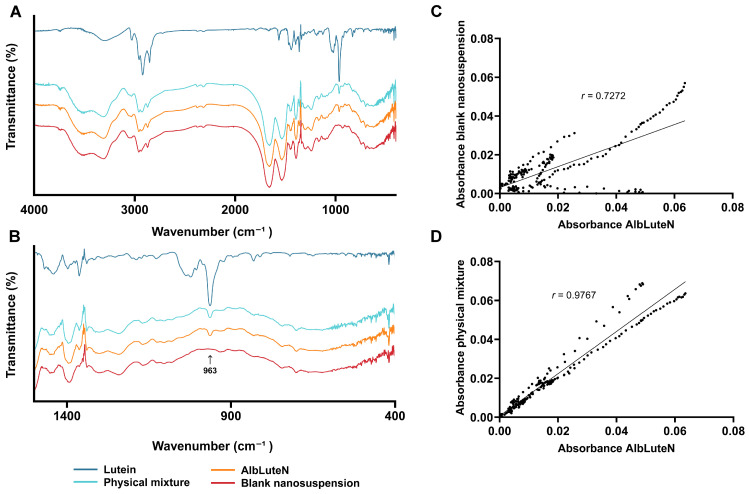
FTIR spectra and comparative analysis. (**A**) Full FTIR spectra of AlbLuteN, the blank nanosuspension, lutein, and the physical mixture of lutein with the blank formulation. (**B**) Fingerprint regions of the corresponding spectra. (**C**) Correlation between AlbLuteN and the blank nanosuspension in the 850–1050 cm^−1^ range. (**D**) Correlation between AlbLuteN and the physical mixture in the 850–1050 cm^−1^ range.

FTIR spectra (Figure 3A) were employed to further assess the encapsulation of lutein within nanoparticles. This evaluation relies on the premise that, if the API is fully entrapped within the nanoparticles, only vibrations corresponding to the surface of the particles, i.e., those associated with the nanoparticle matrix material, should be detectable.

The characteristic absorption bands of lutein were identified using its reference spectrum, which displayed a broad band at 3298 cm^−1^, corresponding to O-H stretching vibrations. Peaks observed at 2921 cm^−1^ and 2952 cm^−1^ were attributed to the asymmetric and symmetric stretching of CH_2_ and CH_3_ groups, respectively. The absorption at 1441 cm^−1^ was associated with CH_2_ scissoring, while the peak at 1363 cm^−1^ corresponded to the splitting of dimethyl groups. Additionally, a distinct peak at 963 cm^−1^ was assigned to the out-of-plane deformation of trans-conjugated alkene (-CH=CH-). These spectral features align with previously reported data [22].

The FTIR spectrum of the blank formulation was analyzed to identify absorption bands characteristic of albumin. The peak at 1536 cm^−1^ was attributed to amide II, corresponding to C-N stretching and N-H bending. Similarly, the peak at 1659 cm^−1^ was assigned to amide I, which is linked to C=O bond vibrations. The presence of CH_2_ groups was confirmed by the band at 1392 cm^−1^, whereas amide III, appearing at 1241 cm^−1^, was associated with C-N stretching and N-H bending. These spectral findings are consistent with literature data [23].

The FTIR spectrum of AlbLuteN exhibited characteristic absorption bands corresponding to albumin, while distinct signals associated with lutein were not clearly observed. However, the spectrum of the physical mixture also did not display well-defined lutein peaks, likely due to its low concentration in the sample. Given that the *DL%* was 4.76 ± 0.39%, the majority of the sample’s mass consisted of albumin, which may explain the low intensity of lutein’s characteristic peaks in the physical mixture. The most intense lutein band, attributed to the out-of-plane deformation of trans-conjugated alkene (-CH=CH-), was located in the fingerprint region at 963 cm^−1^. Focusing the analysis on the fingerprint region (400–1500 cm^−1^) (Figure 3B) revealed a weak peak at 963 cm^−1^ in both the physical mixture and the AlbLuteN sample. To further compare the analyzed sample with the blank formulation and the physical mixture, Pearson correlation coefficients were calculated for spectral absorbance pairs: AlbLuteN vs. blank and AlbLuteN vs. physical mixture. A higher *r* value indicates greater spectral similarity between the compared samples, and *r* = 1 represents a perfect correlation. The correlation coefficient for the full spectra of AlbLuteN and the blank formulation was 0.9986, while that for AlbLuteN and the physical mixture was 0.9994, indicating a very high correlation in both cases. However, when the correlation coefficients were calculated for a narrower spectral range (850–1050 cm^−1^), which included the most intense lutein band, a more pronounced difference in the *r* values was observed: 0.7272 for AlbLuteN vs. the blank (Figure 3C) and 0.9767 for AlbLuteN vs. the physical mixture (Figure 3D). This result suggests a stronger correlation between the AlbLuteN spectrum and that of the physical mixture, implying that lutein is not entirely encapsulated within nanoparticles but is also present outside them, likely forming complexes with albumin.

**Figure 4:**
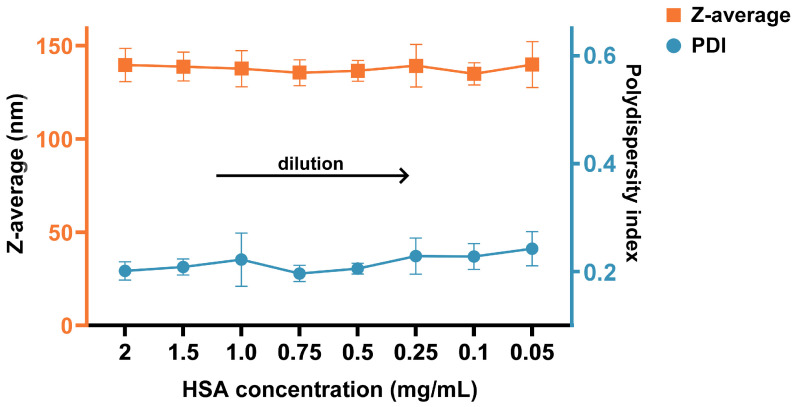
Evaluation of AlbLuteN particle behavior upon dilution. Z-average and PDI were measured at decreasing human serum albumin (HSA) concentrations. Statistical analysis revealed no significant differences in either parameter across all tested dilutions (*p* > 0.05), indicating preserved nanoparticle stability.

The stability of the nanoparticle size distribution upon dilution to a clinically relevant concentration was assessed. Lyophilized AlbLuteN was reconstituted to obtain a concentration of human serum albumin of 2 mg/mL, followed by size distribution measurement. At this concentration, the formulation exhibited a Z-average of 139.7 ± 8.9 nm with a PDI of 0.201 ± 0.017. The sample was then progressively diluted in water for injection, and the Z-average and PDI were reassessed after each dilution step. No significant differences were observed in either parameter (*p* > 0.05) (Figure 4), confirming the stability of particle integrity following dilution.

### 3.2. Parenteral Nutrition Stability Study

No visible phase separation was observed in any of the emulsions, both immediately after preparation and after 24 h. Due to the addition of the intensely orange multivitamin preparation and AlbLuteN, the emulsions initially exhibited a pale yellow color, which remained unchanged over 24 h.

The PFAT5, Z-average, zeta potential, pH, osmolality, and lutein content values for the control and AlbLuteN-supplemented emulsions immediately after preparation, and for AlbLuteN-supplemented emulsions after 24 h are presented in Table 2.

The PFAT5 of all control admixtures was less than or equal to 0.010%. The Z-average of the droplet size of the emulsion ranged from 235.9 ± 2.8 (OPl) to 239.3 ± 2.9 nm (OPe), and the zeta potential ranged from −15.6 ± 0.6 for OSp to −21.6 ± 0.0 mV for OPe. The pH of the control mixtures was within the range of 5.32 ± 0.01 to 5.42 ± 0.01, while the osmolality varied from 918 ± 4 to 1871 ± 8 mOsm/kg.

The PFAT5 of PN admixtures supplemented with AlbLuteN did not increase immediately after sample preparation or within the following 24 h. Interestingly, although all values were low, a downward trend in this parameter was observed after 24 h.

Statistical analysis revealed no significant differences in the Z-average between all control emulsions and those supplemented with AlbLuteN immediately after preparation. Likewise, no significant changes were observed when comparing supplemented emulsions at T_0_ and after 24 h of storage. These results indicate that the addition of AlbLuteN did not alter the Z-averages of the emulsions over the tested period.

The addition of AlbLuteN to the control admixtures did not cause a significant change in zeta potential in any of the emulsions, and the change in zeta potential over 24 h in the supplemented admixtures was statistically significant only in the case of OPl—the value increased from −15.8 ± 0.4 to −15.1 ± 0.3 mV (*p* = 0.0377).

Supplementation with AlbLuteN caused a slight pH change in the admixtures, with further shifts observed after 24 h. The most pronounced pH changes were noted for LPe and OPl—in both cases, the pH increased by 0.07 ± 0.02 units.

AlbLuteN incorporation resulted in osmolality changes within 10 mOsm/kg across the admixtures. After 24 h, a decrease of up to 2 mOsm/kg was observed for LPe, OSp, and OPl. A slightly greater reduction (approximately 10 mOsm/kg) was noted for OPe, corresponding to a change of about 1.09%.

The quantitative analysis revealed the average lutein loss after 24 h to be 2.51 ± 0.95% for LPe, 2.87 ± 6.47% for OPe, and 1.94 ± 6.59% for OSp. In the case of OPl, the lutein content remained stable over the study period, with a slight increase (1.55%), falling within the observed variability in the measurements (±4.67%). This may be attributed to minor differences in extraction efficiency or the dispersion of lutein within the emulsion over time, rather than an increase in its content.

## 4. Discussion

PN requires nutrients to be both soluble and stable in an infusion solution. Lutein, a natural bioactive substance classified as a BCS class II drug [24], exhibits poor aqueous solubility, which limits its administration [25]. To enable its incorporation into PN, the development of a suitable formulation was necessary. For this purpose, we employed nanotechnology, a widely adopted approach to enhance the solubility and bioavailability of hydrophobic compounds [26].

Designing an IV lutein delivery system for PN supplementation required careful consideration. The optimal formulation needed to achieve the following:

- Meet IV drug quality standards to ensure safe clinical application;
- Minimize excipient content to reduce the risk of potential side effects and interactions with PN;
- Ensure a reproducible and scalable formulation process to facilitate large-scale production;
- Efficiently load lutein—the higher the lutein-to-matrix ratio, the lower the total formulation amount required to deliver a given dose, reducing the risk of potential interactions with PN;
- Remain stable post-lyophilization, ideally without requiring additional cryoprotectants that could interfere with PN components.

To obtain an IV formulation of lutein, the nab^TM^ technology was employed—a method used in the production of Abraxane^®^, a human serum albumin-based anticancer formulation of paclitaxel. Like lutein, paclitaxel exhibits poor water solubility, and its conventional IV formulations relied on solubilizers such as Cremophor EL, which is known to cause severe hypersensitivity reactions. The development of Abraxane^®^ eliminated the need for these excipients, thereby reducing adverse effects and enhancing the drug delivery efficiency [12]. By minimizing the excipient content, the nab^TM^ technology improves the safety profile of the lutein formulation, making it better suited for long-term PN use. Furthermore, reducing the excipient load may help to prevent undesirable physical and chemical interactions [27], highlighting the added value of this approach.

The original nab^TM^ technology involves the formation of a pre-emulsion, followed by high-pressure homogenization [13]. We simplified this process by omitting the first step and replacing high-pressure homogenization with sonication. Both methods are established high-energy techniques for producing nanoemulsions with sub-500 nm droplets and narrow size distributions. High-pressure homogenization is widely used in pharmaceutical manufacturing but requires high energy, costly maintenance, and large equipment [28]. In contrast, ultrasound-based emulsification offers high efficiency, process simplicity, and a relatively low cost [29], although it has typically been limited to laboratory-scale applications. Scaling up sonication is challenging due to the reduced cavitation intensity with larger horn sizes. Nevertheless, novel approaches such as the Barbell Horn Ultrasonic Technology have been developed to overcome these limitations, enabling industrial-scale systems to maintain high amplitudes and productivity [28]. Such technologies may facilitate the scale-up of AlbLuteN using advanced sonication systems.

Adick et al. [14] employed sonication to produce albumin-stabilized nanosuspensions for various APIs, using this approach to develop a screening platform to assess the feasibility of incorporating poorly soluble compounds into such a system. Their study confirmed that, if albumin nanoparticles with the desired properties can be obtained by sonication, analogous nanosuspensions can also be successfully prepared using high-pressure homogenization [14]. Based on these findings, AlbLuteN appears suitable for industrial scale-up, not only via sonication but also by adapting the process to high-pressure homogenization. This would require the optimization of key parameters such as the pressure, cycle number, and temperature to ensure reproducible nanoparticle characteristics.

To assess the properties of AlbLuteN and its suitability for parenteral administration, we performed the detailed physicochemical characterization of the formulation. The particle size is a critical parameter in drug delivery systems, influencing the circulation time, biodistribution, and cellular uptake. The preferred size range for IV administration is 2–200 nm, as larger particles are more susceptible to phagocytosis by macrophages, which reduces their permeability and retention [30]. The Z-average of AlbLuteN was 133.5 ± 2.8 nm, placing it within this range. Although the particle size in albumin-based nanosuspensions of different APIs synthesized via sonication varies [14], the Z-average of AlbLuteN closely aligns with that of Abraxane^®^ (approximately 130 nm) [13]. Additionally, our formulation exhibited a PDI of 0.194 ± 0.014, which, although higher than the PDI reported for nab^TM^ paclitaxel (0.11 ± 0.02) [13], still indicates a narrow size distribution [31].

The zeta potential reflects the surface charge of particles and influences their colloidal stability. Typically, values above ± 30 mV provide sufficient electrostatic repulsion to prevent aggregation, while those below ± 10 mV suggest instability [30]. The zeta potential of AlbLuteN was measured at −28.1 ± 1.8 mV, indicating moderate dispersion stability. Compared to Abraxane^®^, which has a reported zeta potential of −24.8 ± 1.6 mV [13], AlbLuteN exhibited a slightly more negative surface charge. A similar trend was observed by Adick et al. [13] for an albumin-stabilized itraconazole nanosuspension, where a more negative zeta potential than Abraxane^®^ was associated with improved colloidal stability.

Although the nab^TM^ technology is promising for poorly water-soluble drugs, its success depends on the API’s physicochemical properties [14]. Our blank nanosuspension lacked uniform particles and showed high heterogeneity and poor reproducibility, underscoring the need for a compatible API. Zeta potential measurements confirmed this observation: AlbLuteN had values indicative of moderate stability, whereas the blank displayed a lower absolute zeta potential (8.47 ± 3.89 mV), suggesting a tendency toward aggregation. These results demonstrate that lutein supports homogeneous nanoparticle formation, making it well suited for this delivery platform.

The successful reconstitution of AlbLuteN following freeze-drying highlights the formulation’s ability to maintain its physicochemical properties without the need for additional cryoprotectants. No significant changes were observed in the Z-average and PDI upon rehydration compared to freshly prepared samples, indicating that nanoparticle aggregation during the freeze-drying process did not occur. Similarly, the zeta potential remained stable after rehydration, confirming that the surface charge of the nanoparticles was unaffected. It is worth noting that AlbLuteN reconstitution is easy and does not require extensive mixing or high-energy processing like sonication, which is important in clinical practice.

Beyond immediate reconstitution, long-term colloidal stability is key for IV feasibility. We monitored redispersed AlbLuteN over five weeks at 4 °C and observed consistent Z-average, PDI, and zeta potential values, indicating no aggregation. Notably, while nab^TM^ paclitaxel is approved for use within 24 h post-reconstitution [32], AlbLuteN remained stable for five weeks, suggesting extended colloidal stability post-reconstitution, which may offer greater flexibility in clinical use.

The pH and osmolality of AlbLuteN reconstituted in 0.9% saline solution are close to physiological levels and fall within the acceptable range for IV administration. Formulations with an extreme pH or high osmolality may damage the endothelium and irritate peripheral veins, increasing the risk of phlebitis. Osmolality exceeding 850 mOsm/kg is generally considered unsuitable for peripheral administration and typically requires central venous access to avoid vascular complications. Conversely, solutions with very low osmolality (<150 mOsm/kg) may cause hemolysis or local irritation [33]. AlbLuteN, with a pH of 6.97 ± 0.01 and osmolality of 328 ± 2 mOsm/kg, falls within the physiologically acceptable range and, if intended for independent IV administration, could be administered via either peripheral or central infusion.

DSC analysis was used to assess the physical state of lutein in AlbLuteN. Pure lutein showed melting peaks at 165 °C and 183 °C, which were also present in the physical mixture with the blank nanosuspension, confirming its crystalline form. These peaks were absent in AlbLuteN, whose thermal profile resembled that of the blank, indicating that lutein was amorphous and molecularly dispersed within the albumin matrix. These findings align with previous reports on albumin-based nanoformulations [14,34,35], where drug amorphization occurred during nanoparticle formation. Since the amorphous state is generally associated with improved solubility [36], this transformation could enhance the dissolution characteristics of lutein in the formulation.

The quantitative analysis of AlbLuteN demonstrated efficient lutein encapsulation, with an *EE%* of 84.4 ± 6.3%. Approximately 15% of lutein was unrecovered, likely due to degradation during processing (e.g., ultrasonication, solvent evaporation, or freeze-drying). The *DL%* of lutein in AlbLuteN reached 4.76 ± 0.39%, which is the maximum given the *EE%*. The proportions of albumin and organic solvent used in this study (1 mL of API solution in dichloromethane per 10 mL of 40 mg/mL human serum albumin solution) align with those typically employed in nab^TM^ technology. Increasing the lutein content in the formulation was not possible due to its limited solubility in dichloromethane. In addition to the *EE%* and *DL%*, we determined the percentage of lutein encapsulated within nanoparticles as 51.4 ± 2.9%. This suggests that approximately half of the total lutein was entrapped within nanoparticles, while the remaining fraction was associated with free albumin in the dispersion. To further investigate lutein entrapment within nanoparticles, we employed FTIR spectroscopy.

FTIR analysis revealed that lutein’s characteristic absorption bands were not distinctly visible in the spectrum of AlbLuteN. This was consistent with the spectra of the blank and its physical mixture with lutein (in a ratio corresponding to that of AlbLuteN), where the lutein peaks were also weak, likely due to the low overall lutein content relative to albumin. However, a focused analysis of a narrower section of the fingerprint region (850–1050 cm^−1^), which included the most intense lutein peak, showed a higher spectral correlation between AlbLuteN and the physical mixture than between AlbLuteN and the blank formulation. Lutein thus appeared to be only partially encapsulated in nanoparticles, with the remainder likely present in a free albumin-bound form. This interpretation is consistent with the HPLC-DAD-based quantitative analysis, which determined that 51.4 ± 2.9% of lutein was encapsulated within nanoparticles. These findings contrast those of previous nab^TM^ formulations obtained by both standard nab^TM^ technology and sonication, where the nearly complete encapsulation (>95%) of the API within nanoparticles has been reported [12,14]. This is likely due to differences in the properties of the active compound or manufacturing processes. The coexistence of both nanoparticle-entrapped and albumin-bound lutein could affect the pharmacokinetic profile of the formulation, potentially enabling biphasic release, combining the immediate bioavailability of the unencapsulated fraction with sustained release from nanoparticles. Such a dual structure may also influence the in vivo distribution profile of lutein and potentially enhance its plasma retention, which could ultimately improve its therapeutic efficacy. A study by Li et al. [12] on the in vivo behavior of albumin-based nanoformulations supports this hypothesis, indicating that API release and biodistribution depend on whether the drug is bound to free albumin or enclosed within particles.

Once infused, Abraxane^®^ nanoparticles undergo extensive dilution in the bloodstream, leading to their disintegration and the release of paclitaxel into a free albumin-bound form. In this state, paclitaxel is efficiently transported into tumors via the gp60 receptor, contributing to targeted delivery and high anticancer activity. However, when albumin nanoparticles remain stable at high dilutions, drug release occurs at a significantly lower rate, and the biodistribution differs from that of free albumin–drug complexes [12]. Adick et al. [13] suggested that nanoparticle stability may depend on the API’s aqueous solubility. In their study, itraconazole-containing dispersions required greater dilution to shift from a nanoparticle-entrapped to an albumin-bound form compared to those with the more water-soluble paclitaxel. To assess the stability of AlbLuteN, we measured the size distribution after dilution in water. No significant changes in the Z-average or PDI were observed down to 0.05 mg albumin/mL. For comparison, Yasuda et al. [17] performed a similar stability study for Abraxane^®^, observing particle disintegration in the range of 0.25–0.1 mg albumin/mL. Although these tests were undertaken in water and represent a simplified model that does not fully replicate the complexity of the biological environment, the comparative results remain informative. Our findings suggest the increased stability of lutein-containing particles compared to those with paclitaxel. This challenges the proposed solubility–stability relationship, as lutein and paclitaxel have similar water solubility (0.26 vs. 0.25 µg/mL, respectively) [22]. Nevertheless, the behavior of AlbLuteN in vivo may differ from that observed in aqueous conditions due to the complex compositions of biological fluids, including proteins and electrolytes. These components could affect nanoparticle stability or protein corona formation. Therefore, additional studies in biologically relevant media or in vivo models would be required to comprehensively evaluate the formulation behavior after administration.

Lutein exhibits antioxidant activity, alleviates hepatic inflammation, and reduces steatosis, suggesting potential to prevent or treat IFALD—a serious PN complication [11]. Given that albumin nanoparticles tend to accumulate in the liver, lungs, and spleen [12], it can be speculated that a portion of the stable, nanoparticle-entrapped lutein may concentrate in the liver, helping to mitigate liver dysfunction associated with IV nutrition. Meanwhile, the unencapsulated albumin-bound fraction could exert systemic antioxidant effects and contribute to ocular protection. However, this hypothesis requires further validation.

Having developed a suitable lutein IV formulation, we proceeded to evaluate the safety of its addition to PN. To identify potential interactions between AlbLuteN and different types of PN admixtures, we tested four commercial PN admixtures with varying compositions: Lipoflex Peri (lacking omega-3 fatty acids) and three Omegaflex variants (Peri, Special, and Plus), which differed in their glucose, amino acid, and electrolyte content. All admixtures were supplemented with multivitamins and trace elements to reflect clinical practice. This allowed us to assess the formulation stability across a range of PN types.

Since lutein is not currently administered intravenously, no established IV dosing guidelines exist. However, lutein is generally recognized as safe (GRAS) [37], and oral supplementation studies frequently use doses ranging from 10 to 20 mg per day [38,39,40]. The European Food Safety Authority has established an acceptable daily intake of 1 mg/kg body weight/day for lutein derived from *Tagetes erecta*, containing at least 80% carotenoids consisting of lutein and zeaxanthin, corresponding to 70 mg/day for a 70 kg adult [41]. Due to the low oral bioavailability of lutein, estimated at approximately 15% [42], a representative IV dose of 10 mg/day (equivalent to ~66 mg administered orally) was selected in our study. In the PN stability assessment, 5 mg of lutein was added to each 1250 mL of the commercial PN admixture, resulting in a total daily dose of 10 mg for patients receiving 2500 mL/day, which is a standard PN volume for individuals weighing approximately 70 kg (35–40 mL/kg body weight/day). Given the absence of clinical experience with IV lutein, this dose was not intended as a therapeutic recommendation but was rather selected to simulate a high-exposure scenario to assess the physicochemical compatibility and potential interaction risk of AlbLuteN when added to PN admixtures.

The safety of PN supplementation with AlbLuteN was evaluated through visual inspection and physicochemical analysis. Freshly prepared samples were compared with lutein-free admixtures to identify immediate interactions. Although minor changes may arise from combining components with different properties, the final formulations must meet established quality criteria. Measurements after 24 h assessed stability over the maximum infusion period. Notable droplet growth or pH shifts indicate emulsion destabilization, which is unacceptable from a patient safety perspective [43].

The infusion of emulsions containing oversized droplets may pose a risk of embolism [44]. Therefore, the particle size is a key quality parameter in IV lipid emulsions and is subject to strict pharmacopeial limits based on two measurement methods [45]. The Z-average must not exceed 500 nm, while the PFAT5 must remain below 0.05%. In our study, the PFAT5 values in all AlbLuteN-supplemented PN admixtures remained well below the pharmacopeial threshold, with no increase observed after 24 h, confirming that the presence of AlbLuteN did not promote coalescence or the formation of larger fat globules. Similarly, the Z-average values remained within the acceptable range, with no significant differences between the control and supplemented emulsions. The absence of changes in droplet size after 24 h indicated that AlbLuteN did not affect the emulsion integrity. Visual inspection confirmed that the emulsions were pale yellow, homogeneous, and remained stable throughout the study. No visible signs of phase separation, discoloration, or instability were observed.

The remaining physicochemical parameters are not governed by pharmacopeial standards; therefore, their interpretation was based on literature data. The principle that dispersions with an absolute zeta potential value exceeding 30 mV exhibit high colloidal stability also applies to IV lipid emulsions [46]. However, the baseline zeta potential values of PN admixtures are often lower due to the presence of various components in the formulations [18]. In our study, control emulsions exhibited zeta potentials ranging from approximately −15 mV in those containing higher amounts of glucose, amino acids, and electrolytes (OSp and OPl) to around −21 mV in peripheral formulations (LPe and OPe) with lower concentrations of these constituents. Supplementation with AlbLuteN did not significantly alter the zeta potential of any formulation. Pronounced changes in pH or osmolality may indicate undesired chemical reactions between PN components [18]. According to the applied acceptance criteria [18], the osmolality should not vary by more than 5%, and more stringent guidelines recommend that pH shifts should not exceed 0.2 units. In our study, none of the admixtures exceeded these thresholds, suggesting that no unexpected interactions occurred between the components.

Another important indicator of PN quality is the stability of the active compound over the infusion period. According to pharmaceutical drug standards, the content of the API should not decrease by more than 10% during the product’s shelf life [19]. In our study, the lutein content in all AlbLuteN-supplemented admixtures decreased by less than 3%, remaining within the acceptable limit. As lutein is highly sensitive to light and oxygen, the results suggest that albumin-based nanosuspension effectively protects it from degradation. Although direct comparison with unformulated lutein was not possible due to its poor solubility, the retention levels support the nanocarrier’s stabilizing potential. Samples were stored at room temperature and protected from light to simulate clinical conditions and assess AlbLuteN’s stability; thus, further studies under varied conditions, such as light exposure, are needed to confirm the protective effect.

This study demonstrates that AlbLuteN can be safely incorporated into the tested PN admixtures without compromising their physicochemical stability, although some limitations should be acknowledged. PN admixtures are highly complex systems whose compositions vary depending on individual patient needs, including the type and ratio of macronutrients, electrolyte levels, and the presence or absence of specific additives. Due to the potential for unpredictable interactions, the compatibility of any co-administered product should ideally be verified within the exact composition intended for clinical use. Our study focused exclusively on lipid-containing admixtures and did not address the compatibility of two-in-one formulations, which may behave differently due to the lack of lipids. As such, the findings cannot be directly generalized to all types of PN regimens. Further research may be needed to confirm these findings across a broader range of formulation types and clinical conditions.

Given the complexity of AlbLuteN as a nanoformulation combining a bioactive compound with a carrier protein for IV administration, the path toward clinical translation is expected to present significant challenges. These include navigating regulatory classification (e.g., as a drug, biologic, or combination product), addressing stringent quality and safety requirements, and defining appropriate clinical endpoints that reflect its role as a supportive agent in PN. Overcoming these challenges will be essential to realizing the therapeutic potential of AlbLuteN in clinical settings. Although the present study provides a comprehensive analysis of the formulation and its physicochemical compatibility within PN systems, further research is warranted to investigate its biological performance prior to clinical application. In particular, future studies should focus on determining the pharmacokinetic profile, biodistribution, and organ-specific accumulation following IV administration. Additionally, the hepatoprotective effect should be validated in a relevant in vivo model. These investigations, together with comprehensive toxicological assessments and evaluations of its immunocompatibility, will be essential to fully establish the safety and therapeutic potential of the formulation in the context of long-term parenteral administration.

## 5. Conclusions

Lutein is a bioactive compound with documented health-promoting properties that is normally obtained through the diet. For patients receiving PN, the dietary intake of such compounds is not possible, and the IV delivery of lutein has so far not been feasible. AlbLuteN, developed in this study, enables the safe addition of lutein to PN admixtures, representing a step toward improving artificial nutrition by partially bridging the gap between IV feeding and the composition of a balanced diet. While further pharmaceutical development would be required for AlbLuteN to be considered a drug product, our findings support its compatibility with representative PN formulations. The next step will involve biological studies to explore the therapeutic potential of lutein delivered in this system, particularly in the context of IFALD, one of the most serious complications of long-term PN. The albumin-based nanoformulation described here may also offer a platform for the delivery of other bioactives in PN, particularly those with poor aqueous solubility or limited stability.

## Figures and Tables

**Table 1:** Characteristics of PN admixtures.

Parameter/Component	Unit	LPe	OPe	OSp	OPl
Volume	mL	1250	1250	1250	1250
Glucose	g	80	80	180	150
Amino acids		40	40	70.1	48
Soybean oil		25	20	20	20
MCT		25	25	25	25
Omega-3 TG		0	5	5	5
Total lipids		50	50	50	50
Sodium	mmol	50	50	67	50
Potassium		30	30	47	35
Magnesium		3.0	3.0	5.3	4.0
Calcium		3.0	3.0	5.3	4.0
Phosphates		7.5	7.5	20	15
Total energy	kcal	955	955	1475	1265
Theoretical osmolarity	mOsm/kg	840	840	1545	1215

LPe, Lipoflex Peri; OPe, Omegaflex Peri; OSp, Omegaflex Special; OPl, Omegaflex Plus; MCT, medium-chain triglycerides; Omega-3 TG, triglycerides of omega-3 fatty acids.

**Table 2:** Physicochemical parameters of control samples immediately after preparation and AlbLuteN-loaded samples immediately after preparation and after 24 h.

PN Admixture	PFAT5 * (%)	Z-Average (nm)	Zeta Potential (mV)	pH	Osmolality (mOsm/kg)	Lutein Content (%)
LPe control (T_0_)	0.002	238.9 ± 2.8	−21.6 ± 0.3	5.37 ± 0.01	933 ± 9	N/A
LPe + AlbLuteN (T_0_)	0.002	244.5 ± 0.3	−21.3 ± 0.5	5.36 ± 0.01	925 ± 5	100.00 ± 0.73
LPe + AlbLuteN (T_24_)	0.001	245.3 ± 0.5	−20.8 ± 0.2	5.43 ± 0.02	924 ± 1	97.49 ± 0.61
OPe control (T_0_)	0.010	239.3 ± 2.9	−21.6 ± 0.0	5.32 ± 0.01	918 ± 4	N/A
OPe + AlbLuteN (T_0_)	0.008	237.3 ± 0.8	−21.1 ± 0.9	5.37 ± 0.01	921 ± 4	100.00 ± 1.80
OPe + AlbLuteN (T_24_)	0.005	240.0 ± 0.9	−20.7 ± 0.0	5.42 ± 0.01	911 ± 16	97.13 ± 6.21
OSp control (T_0_)	0.001	237.3 ± 2.7	−15.6 ± 0.6	5.42 ± 0.01	1871 ± 8	N/A
OSp + AlbLuteN (T_0_)	0.001	239.9 ± 1.3	−16.0 ± 0.6	5.43 ± 0.02	1865 ± 6	100.00 ± 6.04
OSp + AlbLuteN (T_24_)	0.000	241.7 ± 2.0	−16.0 ± 0.2	5.49 ± 0.01	1863 ± 7	98.06 ± 2.64
OPl control (T_0_)	0.004	235.9 ± 2.8	−15.9 ± 0.3	5.35 ± 0.01	1424 ± 2	N/A
OPl + AlbLuteN (T_0_)	0.004	239.4 ± 2.4	−15.8 ± 0.4	5.35 ± 0.00	1414 ± 6	100.00 ± 3.51
OPl + AlbLuteN (T_24_)	0.001	240.0 ± 1.8	−15.1 ± 0.3	5.42 ± 0.02	1412 ± 2	101.55 ± 3.08

* SD < 0.001; LPe, Lipoflex Peri; OPe, Omegaflex Peri; OSp, Omegaflex Special; OPl, Omegaflex Plus; T_0_, immediately after sample preparation; T_24_, after 24 h of storage; N/A, not applicable.

## Data Availability

The raw data supporting the conclusions of this article will be made available by the authors on request.

## Abbreviations

The following abbreviations are used in this manuscript:

PN	Parenteral nutrition
AlbLuteN	Albumin–lutein nanosuspension
PFAT5	Percentage of fat residing in globules larger than 5 µm
Z-average	Intensity-weighted mean hydrodynamic diameter
IV	Intravenous
IFALD	Intestinal failure-associated liver disease
nab^TM^	Nanoparticle albumin-bound
API	Active pharmaceutical ingredient
FDA	Food and Drug Administration
HPLC	High-performance liquid chromatography
PDI	Polydispersity index
DSC	Differential scanning calorimetry
DAD	Diode-array detection
DL%	Drug loading
EE%	Entrapment efficiency
NL%	Nanoparticle lutein
FTIR	Fourier transform infrared
LPe	Lipoflex Peri
OPe	Omegaflex Peri
OSp	Omegaflex Special
OPl	Omegaflex Plus
T_0_	Immediately after sample preparation
T_24_	After 24 h of storage
BCS	Biopharmaceutical classification system
GRAS	Generally recognized as safe
